# Clinical course of patients with episodic cluster headache treated with corticosteroids inproximity to the sphenopalatine ganglion: A preliminary study of 23 patients

**DOI:** 10.4317/medoral.17578

**Published:** 2011-12-06

**Authors:** Maria Peñarrocha-Diago, Araceli Boronat, David Peñarrocha-Oltra, Javier Ata-Ali, Jose V. Bagan, Miguel Peñarrocha-Diago

**Affiliations:** 1 Associate Professor of Oral Surgery. Valencia University Medical and Dental School; 2 DDS. Master in Oral Surgery and Implantology. Valencia University Medical and Dental School; 3 DDS. Resident of the Master in Oral Surgery and Implantology. Valencia University Medical and Dental School; 4 DDS. Primary Public Health Service Dentist. Valencian Health Service. Master in Oral Surgery and Medicine. Master in Oral Surgery and Implantology. Valencia University Medical and Dental School; 5 Chairman of Oral Medicine. Valencia University Medical and Dental School. Head of the Department of Stomatology. Valencia University General Hospital; 6 Chairman of Oral Surgery. Director of the Master in Oral Surgery and Implantology. Valencia University Medical and Dental School. Valencia (Spain)

## Abstract

Objective: A study is made of the clinical course of patients with episodic cluster headache following the injection of corticosteroids in the proximity of the sphenopalatine ganglion of the affected side. Study Design: A retrospective observation study was made corresponding to the period between 2006 and 2010. Patients with episodic cluster headache received corticosteroid infiltrations in the vicinity of the sphenopalatine ganglion. Data were collected to assess the clinical course, quantifying pain intensity and quality of life. A total of 23 patients (11 women and 12 men) with a mean age of 50.4 years (range 25-65) were included. Forty percent of the patients had undergone dental extractions in the quadrant affected by the pain, before the development of episodic cluster headache, and 37.8% underwent extractions in the same quadrant after appearance of the headache. Results: Most of the patients suffered 1-3 attacks a day, with a duration of pain of between 31-90 minutes. The mean pain intensity score during the attacks at the time of the first visit was 8.8 (range 6-10), versus 5.4 (range 3-9) one week after the first corticosteroid injection. On the first visit, 86.9% of the patients reported unbearable pain, versus 21.7% after one week, and a single patient after one month. Conclusions: The evolution of episodic cluster headache is unpredictable and variable, though corticosteroid administration clearly reduces the attacks and their duration.

** Key words:**Episodic cluster headache, vascular pain, sphenopalatine ganglion, corticosteroid infiltration.

## Introduction

Episodic cluster headache (ECH) is characterized by unilateral attacks of pain in the upper premolar, periorbital or temporal region, or in a combination of these locations, accompanied by ipsilateral conjunctiva injection, tearing, nasal congestion, perspiration of the affected side of the face, mitosis, and homolateral palpebral ptosis and edema. The attacks typically last 15 to 180 minutes, and patients experience one or several attacks a day in the course of an outbreak of variable duration (weeks or months). The episodic presentation of the disorder is characterized by periods of complete remission (1,2).

The etiopathogenesis of cluster headache is unclear, but appears to involve the activation of parasympathetic nerve structures in the sphenopalatine ganglion, which would explain many of the associated symptoms. In addition, activation of the ipsilateral hypothalamic gray matter could explain the typical circadian and circumannual periodicity of the disorder (3). The sphenopalatine ganglion is the largest peripheral parasympathetic ganglion with connections to the sensory fibers and plexus of the internal carotid artery (4). The sensory and motor fibers and autonomic components are implicated in the physiopathology ECH (5). In the case of chronic headaches resistant to drug therapy, sphenopalatine ganglion block has been shown to offer some efficacy (3). The most common indications of sphenopalatine ganglion block include cluster headache and atypical facial pain (6).

Treatment efficacy in cluster headache is difficult to evaluate, due to the great variations in the duration of each outbreak. Until improved knowledge is gained of the etiopathogenic mechanisms of cluster headache, the most useful treatment option is to counter one of the phenomena giving rise to the pain: arterial vasodilatation. The management of ECH includes three treatment modalities: non-pharmacological (general measures and patient education), pharmacological (abortive drugs and preventive treatment) and surgical.

Abortive therapy aims to arrest individual attacks, and comprises the use of subcutaneous sumatriptan, ergotamine and oxygen inhalation (2,7). The aim of preventive treatment is to shorten the duration of ECH and reduce the frequency of the attacks. In this case corticosteroids, verapamil, lithium, topiramate and greater occipital nerve block have been found to be effective (2,8,9). There is clinical evidence of the efficacy of corticosteroids in application to cluster headache (10). In effect, these drugs quickly improve the attacks in both the chronic and episodic forms of the disease, as a result of their central and peripheral antiinflammatory effects (11).

The present study evaluates the clinical course of patients with episodic cluster headache following the injection of corticosteroids in the proximity of the sphenopalatine ganglion of the affected side.

## Material and Methods

Patient selection

A retrospective, descriptive and follow-up clinical case study was carried out between the years 2006 and 2010 in patients with episodic cluster headache seen in a University oral surgery clinic. Episodic cluster headache (ECH) was diagnosed according to the criteria of the International Headache Society (HIS) (1): 1) severe or very severe unilateral pain affecting the orbital, supraorbital, temporal and/or upper maxillary regions, with a duration of 15 to 180 minutes; 2) headache accompanied by signs of autonomic dysfunction; 3) pain characterized by a frequency of between one attack every two days and 8 attacks a day; and 4) pain not attributable to any other disease.

The following patient inclusion criteria were used: 1) attacks of cluster headache; 2) treatment with corticosteroid injections close to the sphenopalatine ganglion; and 3) a minimum follow-up of 12 months. The criteria used for corticosteroid injection were: 1) patients with active ECH even after the start of some other treatment; and 2) the absence of general contraindications to corticosteroid injection (diabetes or gastrointestinal ulcer). Episodic cluster headache was finally diagnosed in 26 patients; three patients were excluded due to a clack of the required minimum 12 months of follow-up. We thus included 23 patients with ECH, with a mean age of 50.4 years (range 25-65) (11 women and 12 men). Forty percent of the patients had undergone dental extractions in the quadrant affected by the pain, before the development of episodic cluster headache, and 37.8% underwent extractions in the same quadrant after appearance of the headache.

Data collection and follow-up of pain

The following data were collected: patient age and gender, dental surgical treatment in the affected quadrant before and after appearance of the pain, and characteristics of the pain: location, frequency (number of attacks/day), duration of the attacks (minutes), and intensity (based on a 10-cm visual analog scale (VAS), where 10 corresponds to maximum pain intensity).

Patient disability and quality of life were evaluated by means of a clinical scale scored from 0-6, where 1 = no pain, 2 = mild pain (pain attacks some days of the month, with practically no impact upon quality of life), 3 = bothersome pain (pain attacks some days of the month, causing concern, but with almost normal daily life activities), 4 = moderate pain (occasional daily attacks or pain at some point during the day, preventing normal live), 5 = important pain (frequent daily attacks seriously affecting quality of life) and 6 = terrible pain (very frequent daily attacks very seriously affecting quality of life). We documented the days with pain corresponding to the period from the last outbreak to the time of the first visit (less than 7 days, 7-15 days and over 15 days), and the autonomic manifestations associated with the pain.

The patients received corticosteroid injections in the proximity of the sphenopalatine ganglion of the affected side. In the case of more than one attack a day, we prescribed prophylactic treatment in the form of 2 mg of ergotamine via the oral route. In patients with a single daily attack of pain we only administered corticosteroids in the vicinity of the sphenopalatine ganglion. One infiltration a week was carried out during four weeks, and in the absence of pain throughout the week, no further infiltrations were made. The possible side effects of the medication were recorded.

Injection technique

Truncal superior posterior dental nerve block was carried out using an auto-aspirating metal syringe and long needle (25-30 mm) with 4% articaine and adrenalin 1:100,000 (Inibsa®, Lliça de Vall, Barcelona, Spain). The injection site was on the posterolateral surface of the upper maxilla, at 1 cm above and behind the last molar. The needle penetrated mesial to the upper first molar and was advanced towards the zygomatic process until reaching the vestibular depth of the upper second molar, directing inwards, upwards and backwards to form an angle of approximately 45 degrees with respect to the upper occlusal plane. The anesthetic solution was slowly injected after negative aspiration.

While waiting a few minutes for the anesthetic solution to exert its effect, the empty anesthetic carpule was filled with 40 mg of triamcinolone acetonide (Trigon Depot®, Bristol-Meyers Squibb, Madrid, Spain), and the full contents of the carpule were injected into the vicinity of the sphenopalatine ganglion (in the same way as the anesthetic block).

Clinical course 

The patient clinical course and improvement were assessed according to the pain intensity (on the first visit, every week after infiltration, and after one month) and the clinical disability scale (on the first visit, after 1, 3 and 6 months, and after one year). The duration of the outbreak was classified as follows: less than 10 days, 11-20 days, 21-40 days, 41-60 days and over 60 days.

## Results

In 47.8% of the patients the pain was located in the premolar region, irradiating towards the orbit and temporal region; in 39.1% of the cases the pain was located in the periorbital region, irradiating towards the temple; and in 13% of the subjects the pain was diffusely located in the upper maxillary, temporal and mandibular region. The pain affected the right side in 56.5% of the patients, and the left side in 43.5%. Most of the subjects experienced between 1-3 attacks/day, and in most cases the attacks lasted 31-60 minutes ( Table 1).

**Table 1:**
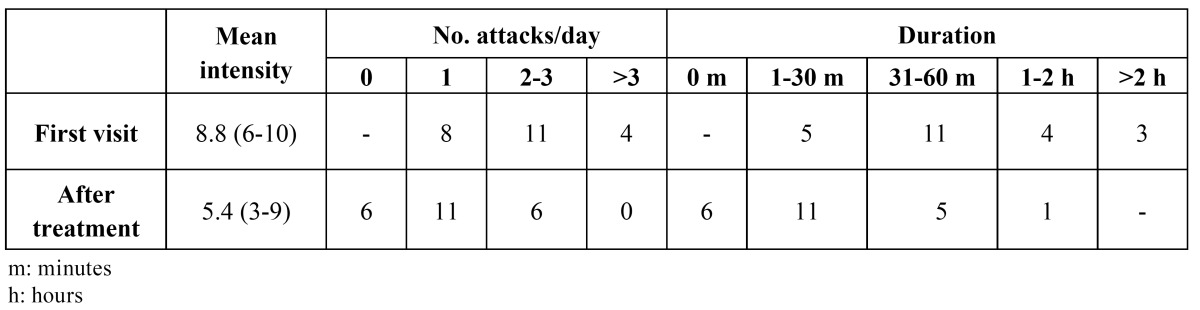
Number of attacks and pain intensity and duration before treatment.

The pain had been present for under 10 days in 26% of the patients, between 11-20 days in 17.3%, between 21-40 days in 13%, between 41-60 days in 17.3%, and for over 60 days in 26% of the patients. The mean intensity of pain on the first visit was 8.8 (range 6-10). The clinical scale on the first visit showed patient disability to be moderate in 13% of the case, important in 56.6%, and terrible in 30.4%. The pain was accompanied by vegetative manifestations in 73% of the cases – particularly nasal secretion, tearing and reddening in the affected zone.

Corticosteroid treatment involved a single infiltration in 26% of the cases, two injections in 52.1%, and three injections in 21.7%. A total of 73.9% of the patients received additional drug treatment.

The data relating to the number and duration of the attacks after the treatment received is reported in ( Table 1). One week after the first injection, the mean pain intensity score was 5.4 (range 3-9), while one week after the second injection the mean score was 4.4 (range 2-9). In turn, after the third injection the mean pain intensity was 4.6 (range 3-9), versus 2.8 (range 0-7) after one month. ( Table 2) describes the evolution of patient disability. On the first visit, 86.9% of the patients suffered unbearable pain, versus 21.7% after one week, and a single patient after one month. The patients reported marked improvement of the intensity of the attacks

**Table 2:**
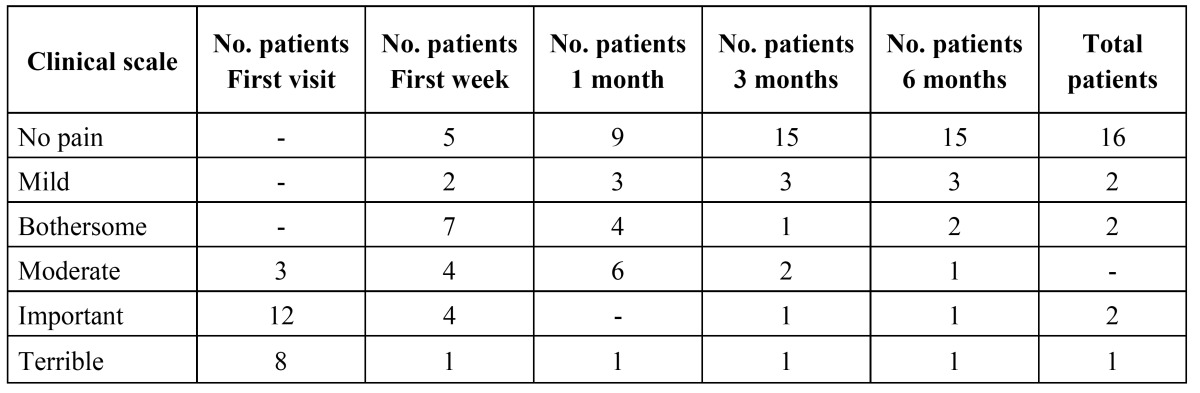
Clinical scale assessing patient disability according to pain.

(Fig. 1). After 12 months, 69.6% of the patients were free of pain, with or without medication; 17.4% showed clear improvement; and 13% experienced no significant improvement. The duration of the outbreak was under 10 days in 26% of the cases, 11-21 days in 17.3%, 21-40 days in 13%, 41-60 days in 17.3%, and over 60 days in 26%. None of the patients experienced adverse effects as a result of the medication provided.

**Figure 1:**
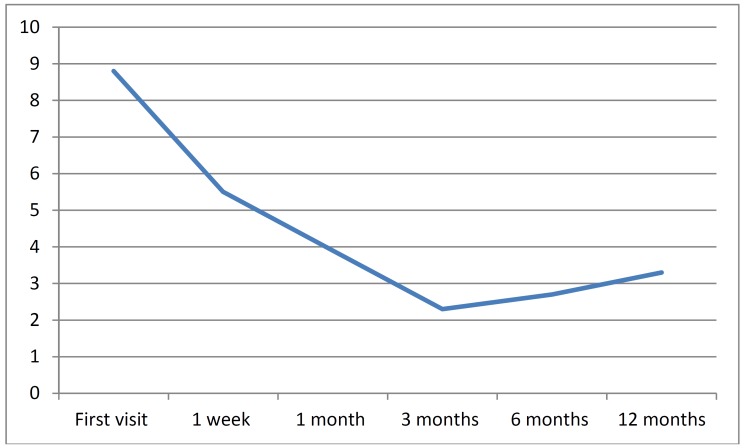
Evolution of pain following the corticosteroid infiltrations.

## Discussion

Episodic cluster headache usually manifests in the second or third decade of life, though it can also appear in older age groups. In effect, the literature describes cases of first attacks of ECH manifesting within the 8-62 years age range (12). In our study the mean patient age was 50.4 years (range 25-65). Regarding patient gender distribution, most of our patients were males, in coincidence with the findings in the literature (12). However, according to Manzoni (13), the male predominance is gradually decreasing – possibly as a result of changes in lifestyle in both sexes, particularly in women (14).

Many patients with cluster headache are wrongly diagnosed with symptomatic headache due to dental disease – this often leading to unnecessary and ineffective dental treatments (15). Bahra and Goadsby (16) found that 230 out of 511 patients had been examined by a dentist before being diagnosed with cluster headache. Leroux and Ducros (17) in turn found 16% of the patients to be subjected to dental or ocular treatment, or sinus surgery. In our series, 37.8% of the patients underwent dental extractions in the affected quadrant after appearance of the pain, and posteriorly some of them requested even further dental treatments. In the study of Bittar and Graff-Radford (18), 14 out of 33 patients with cluster headache underwent some type of invasive and irreversible dental procedure.

The pain is located in the periorbital and temporal area, extending to the ear and even to the occipital-cervical region (17). In the present study, the most common location was the region of the premolars and the periocular zone, in coincidence with the findings of most authors (15,19). This in turn led to dental extractions in 40% of the cases, in an attempt to resolve the pain, and extractions were even carried out in 37.8% of the cases after cluster headache had been diagnosed.

Most patients experience 1-3 attacks a day (19). According to Russell et al. (20), the total duration of the attacks was under one hour in 78% of the cases – this being similar to the duration recorded in our study (70.8%). Autonomic signs and symptoms are included among the diagnostic criteria of cluster headache (1). In this context, Nappi et al. (21) recorded vegetative manifestations in 97% of all cases, while in our series 78% of the patients suffered such manifestations, which disappeared with resolution of the pain.

A variety of treatments have been investigated for prevention or symptoms relief in these patients, including triptans (22,23), ergotamine (24), topiramate (8), hyperbaric oxygen therapy (25), and oral or parenteral corticosteroids (10,26,27). Surgery must be viewed with great caution, since there are no reliable long-term data on the results obtained. In this context, different techniques have been proposed, such as radiofrequency ablation (28), rhizotomy of Gasser’s ganglion (29), greater occipital nerve block (9), and sphenopalatine ganglion block (3,4).

Mir et al. (27) administered 250 mg of methylprednisolone as an intravenous bolus dose during three consecutive days, followed by 90 mg/day via the oral route for the next 10 days. With this treatment the attacks decreased significantly (p<0.05) compared with patients receiving other medications. In our study the infiltration of 40 mg of triamcinolone acetonide in the proximity of the sphenopalatine ganglion was found to be effective and safe, though in coincidence with other authors, the underlying mechanism of action is not clear (26).

According to Torelli and Manzoni (30), 85.7% of the patients exhibit a peak pain intensity score of between the 8 and 10 on the visual analog scale. In our study the mean pain score on the first visit was 8.8, and corticosteroid injection resulted in a reduction of the frequency and intensity of the attacks. On the first visit, 86.9% of the patients reported unbearable pain, versus 21.7% after one week, and a single patient after one month. No data for comparison are found in the literature. In any case, although it is very difficult to establish the natural course of patients with ECH, clear improvement was noted. It is difficult to establish a homogeneous sample, since the patients present subjective pain of different intensity and behavior over time. Controlled and randomized studies are needed to firmly establish the role of corticosteroids administered in the vicinity of the sphenopalatine ganglion in these patients.

The evolution of the patients with episodic cluster headache is unpredictable and variable, though the administration of corticosteroids clearly reduced the duration and intensity of the pain attacks, and the duration of the outbreaks.
